# Between two storms, vasoactive peptides or bradykinin underlie severity of COVID‐19?

**DOI:** 10.14814/phy2.14796

**Published:** 2021-03-09

**Authors:** Vardan T. Karamyan

**Affiliations:** ^1^ Department of Pharmaceutical Sciences and Center for Blood Brain Barrier Research School of Pharmacy TTUHSC Amarillo TX USA

**Keywords:** cytokine, inflammation, neurolysin, neurotensin, substance P, vascular permeability

## Abstract

Coronavirus disease 2019 (COVID‐19), caused by severe acute respiratory syndrome coronavirus 2 (SARS‐CoV‐2), continues to be a world‐wide pandemic with overwhelming socioeconomic impact. Since inflammation is one of the major causes of COVID‐19 complications, the associated molecular mechanisms have been the focus of many studies to better understand this disease and develop improved treatments for patients contracting SARS‐CoV‐2. Among these, strong emphasis has been placed on pro‐inflammatory cytokines, associating severity of COVID‐19 with so‐called “cytokine storm.” More recently, peptide bradykinin, its dysregulated signaling or “bradykinin storm,” has emerged as a primary mechanism to explain COVID‐19‐related complications. Unfortunately, this important development may not fully capture the main molecular players that underlie the disease severity. To this end, in this focused review, several lines of evidence are provided to suggest that in addition to bradykinin, two closely related vasoactive peptides, substance P and neurotensin, are also likely to drive microvascular permeability and inflammation, and be responsible for development of COVID‐19 pathology. Furthermore, based on published experimental observations, it is postulated that in addition to ACE and neprilysin, peptidase neurolysin (Nln) is also likely to contribute to accumulation of bradykinin, substance P and neurotensin, and progression of the disease. In conclusion, it is proposed that “vasoactive peptide storm” may underlie severity of COVID‐19 and that simultaneous inhibition of all three peptidergic systems could be therapeutically more advantageous rather than modulation of any single mechanism alone.

## 1 INTRODUCTION

The COVID‐19 world‐wide pandemic continues to have devastating socioeconomic impact with limited therapeutic options. As for any disease, in case of COVID‐19 too, detailed understanding of pathogenic mechanisms is critical for development of new therapies. Hence, it is natural that researchers around the world have used various approaches to explore and understand the COVID‐19 morbidity (Sparks et al., 2020; Wang et al., 2020). Among these, arguably most notable are studies focusing on inflammatory mechanisms, pro‐inflammatory cytokines, and “cytokine storm,” since they are fundamentally associated with progression of the disease and mortality of COVID‐19 patients (Mahmudpour et al., 2020; Rossi et al., 2020).

By now, it is well‐recognized that the entry of SARS‐CoV‐2 to the host cell is mediated by peptidase ACE2 (angiotensin converting enzyme 2 (Hoffmann et al., 2020)), which is an important member of the renin‐angiotensin system (RAS) primarily responsible for conversion of angiotensin II into angiotensin‐(1‐7) (Karamyan & Speth, 2007a; Xia & Lazartigues, 2010). This link between SARS‐CoV‐2 and ACE2 has led to many experimental and clinical studies to explore the potential alteration of the RAS function in this disease, extend understanding of the pathology and guide the use of RAS‐modulating drugs in COVID‐19 patients (Sparks et al., 2020; Speth, 2020). Notably, a smaller number of investigators have recognized the intricate association of another peptidergic system, that is, bradykinin or kallikrein‐kinin system, with the RAS within the context of COVID‐19 (Ghahestani et al., 2020; Roche & Roche, 2020; van de Veerdonk et al., 2020). Since angiotensin converting enzyme (ACE), which converts angiotensin I into angiotensin II (Karamyan & Speth, 2007a; Moraes et al., 2017; Speth & Karamyan, 2008), is also central in degradation of bradykinin (Byrd et al., 2006; Israili & Hall, 1992), alteration of the RAS function in COVID‐19 could also mean changed activity of the bradykinin system. This was timely recognized by several groups of investigators early during the pandemic (Roche & Roche, 2020; van de Veerdonk et al., 2020) and most recently was confirmed by a preliminary gene expression analysis study focusing on detailed evaluation of the RAS members, bradykinin and associated systems in samples from COVID‐19 and control patients (Garvin et al., 2020). This is a critical development, because bradykinin is a potent inflammatory mediator that has been associated with a number of pathophysiological conditions including angioedema (Patel & Pongracic, 2019), vasculitis (Karpman & Kahn, 2009), asthma (Ricciardolo et al., 2018), autoimmunity (Dutra, 2017), acute brain injury and neuroinflammation (Albert‐Weissenberger et al., 2013).

Both the original proposals about the potential involvement of the bradykinin system in COVID‐19 pathology (Roche & Roche, 2020; van de Veerdonk et al., 2020) and the recent gene expression analysis study conducted by Garvin and colleagues (Garvin et al., 2020) did an elegant job in explaining the close association of bradykinin with the RAS and linking it to many of the COVID‐19 outcomes. The proposed “bradykinin storm” hypothesis by Garvin and colleagues (Garvin et al., 2020) is an intriguing and welcome development in deciphering the pathogenic mechanisms of COVID‐19. Unfortunately, the presented picture may not be complete, since these investigators did not account for two other major peptidergic systems, substance P and neurotensin, which are also inactivated by ACE and are potent mediators of microvascular permeability, edema formation and inflammation—mechanisms that are in the core of the “bradykinin storm” hypothesis. The need to take into consideration involvement of substance P and neurotensin is further emphasized by Garvin et al. data suggesting downregulation of another peptidase neprilysin in COVID‐19 patients, since this enzyme also, similar to ACE, inactivates bradykinin, substance P and neurotensin. Therefore, in this focused review manuscript the most relevant published studies are summarized to provide evidence about the potential involvement of substance P and neurotensin, in addition to bradykinin, in pathogenic mechanisms of COVID‐19. Furthermore, a case is made that another peptidase known as neurolysin (Nln) is also likely to be affected in this disorder. Based on this, it is proposed that perhaps “vasoactive peptide storm” rather than “bradykinin storm” underlies many of the COVID‐19 outcomes. Lastly, potential therapeutic avenues that could be used to modulate the “vasoactive peptide storm” are discussed.

Since the investigative teams proposing involvement of the bradykinin system in COVID‐19 pathology (Garvin et al., 2020; Roche & Roche, 2020; van de Veerdonk et al., 2020) did a credible job in detailing the basics of the bradykinin and RAS systems, these will not be the primary focus of the present manuscript. Likewise, the readers are advised to refer to several excellent reviews covering the general function and role of the substance P and neurotensin systems in physiological and pathophysiological conditions (Datar et al., 2004; Kleczkowska & Lipkowski, 2013; Mustain et al., 2011; Onaga, 2014).

## 2 ACE AND NEPRILYSIN IN DEGRADATION OF BRADYKININ, SUBSTANCE P, AND NEUROTENSIN

Among the most recognized examples of adverse side effects afforded by ACE inhibitors are cough (experienced by 5–20% of patients) and angioedema (experienced by 0.1–0.5% of patients), both of which are associated with elevated levels of bradykinin and substance P in patients taking these drugs (Byrd et al., 2006; Israili & Hall, 1992). This is because, in addition to conversion of angiotensin I to angiotensin II, ACE also inactivates bradykinin and substance P. Notably, a recent gene expression analysis by Garvin and colleagues has documented downregulation of ACE (Speth, 2020) in COVID‐19 patients (Garvin et al., 2020) suggesting that not only bradykinin but also the levels of substance P could be elevated. This idea is further supported by substantially decreased expression of peptidase neprilysin, otherwise known as neutral endopeptidase in the same COVID‐19 patient samples (Garvin et al., 2020), since this peptidase also degrades and inactivates bradykinin and substance P (Scholzen & Luger, 2004; Skidgel & Erdos, 2004). Importantly, this is the main reason that development of a dual ACE/neprilysin inhibitor omapatrilat was halted, because the use of this drug was associated with considerably higher incidence of angioedema in patients with cardiovascular disorders (Campbell, 2018; Sulpizio et al., 2005).

Within the context of bradykinin and substance P, and their pro‐inflammatory actions, it is important to recognize that that another bioactive peptide neurotensin is also degraded by ACE and neprilysin (Kanellopoulos et al., 2020; Skidgel & Erdos, 2004). However, the clinical significance of elevated neurotensin levels in response to inhibition of ACE or neprilysin has not been systematically studied.

## 3 MICROVASCULAR PERMEABILITY INDUCED BY BRADYKININ, SUBSTANCE P, AND NEUROTENSIN

The role of bradykinin in inducing microvascular permeability was well described by all three investigative teams linking this peptide to the pathogenic mechanisms of COVID‐19 (Garvin et al., 2020; Roche & Roche, 2020; van de Veerdonk et al., 2020). Nevertheless, there is a large body of literature indicating that substance P and neurotensin also facilitate microvascular hyperpermeability. For example, numerous experimental studies documented the ability of substance P and neurotensin to induce microvascular permeability, during intraarterial/‐venous administration, in different vascular beds including skin and mucosal tissues (Gyorfi et al., 1995; Inoue et al., 1996), gastrointestinal tract (Figini et al., 1997; Harper et al., 1984), lungs (Gitter et al., 1995; Rioux et al., 1983), and dura mater (Dux & Messlinger, 2001; Ghabriel et al., 1999). Substance P and neurotensin exert these effects primarily through their respective NK‐1 (neurokinin‐1 receptor) and NTR1 (neurotensin receptor 1) receptors; however, involvement of other receptor subtypes is also possible. Notably, analogous to bradykinin, a number of endogenous modulators and signaling molecules, released from endothelial or other cells, have been described to play a role in substance P and neurotensin‐induced microvascular hyperpermeability, including but not limited to histamine, prostaglandins, nitric oxide, and VEGF (Carraway et al., 1982; Johnson et al., 2016; Katsanos et al., 2008; Osadchii, 2015; Shaik‐Dasthagirisaheb et al., 2013). Our recent study carried out in human, induced pluripotent stem cell (iPSC)‐derived brain microvascular endothelial cells (i.e., *in vitro* model of blood–brain barrier, BBB) adds to these observations by documenting increased BBB permeability in response to substance P, neurotensin, and bradykinin (Al‐Ahmad et al., 2021). Importantly, combination of these peptides at sub‐effective concentrations also resulted in increased BBB permeability in this study indicating that substance P, neurotensin, and bradykinin can potentiate each other's effects at conditions when all three peptides are available concurrently.

It is noteworthy, that the enhanced microvascular permeability induced by these peptides is directly linked to formation of edema in pathophysiological conditions (Castagliuolo et al., 1999; Donelan et al., 2006). Among these, perhaps best‐documented are ACE inhibitor‐induced angioedema (Byrd et al., 2006; Campbell, 2018) and pathogenic BBB opening and edema formation during acute neurodegenerative disorders such as stroke and traumatic brain injury (Donkin et al., 2009; Groger et al., 2005; Jayaraman et al., 2020; Sorby‐Adams et al., 2019; Trabold et al., 2010).

## 4 INFLAMMATION POTENTIATED BY BRADYKININ, SUBSTANCE P, AND NEUROTENSIN

As discussed earlier for bradykinin (Garvin et al., 2020; Roche & Roche, 2020; van de Veerdonk et al., 2020), both substance P and neurotensin are also well‐known for their pro‐inflammatory actions. Many of these effects are linked to the ability of substance P and neurotensin to stimulate release of pro‐inflammatory cytokines, matrix metalloproteases, and other mediators from various cell types, including immune and mast cells (Alysandratos et al., 2012; Coll et al., 2020; Donelan et al., 2006; Miller et al., 1995; Theoharides et al., 1998; Xiao et al., 2018; Yano et al., 1989). Additionally, enhanced expression of cell adhesion molecules, including CD44, in response to substance P and neurotensin has been documented in different cells (Meyer‐Siegler & Vera, 2005; Robbins et al., 1995; Shahrokhi et al., 2010). Similar to microvascular permeability, these effects are mainly mediated through NK‐1 and NTR1 receptors, however there is experimental evidence suggesting the potential involvement of other receptors (e.g., MRGPRX2 for substance P and NTR3/sortilin for neurotensin) in transducing some of these effects (Green et al., 2019; Patel et al., 2016). It is important to note that these effects of substance P and bradykinin are often associated with formation of edema and pain sensation (classic symptoms of inflammation), and have been observed in both peripheral tissues and the CNS (Katsanos et al., 2008; O'Connor et al., 2004; Saiyasit et al., 2020; Sorby‐Adams et al., 2017; St‐Gelais et al., 2006; Suvas, 2017).

Similar to microvascular permeability and edema formation, the role of substance P and bradykinin in neurogenic inflammation and worsening of disease outcomes has been demonstrated in experimental models of stroke and traumatic brain injury (Albert‐Weissenberger et al., 2013; Corrigan et al., 2016). It should be noted, that evidence on direct involvement of neurotensin in neurogenic inflammation in acute brain injury setting is more limited, however one study linked the increased levels of neurotensin as well as substance P and bradykinin in ischemic brain tissue with aggravated stroke outcomes (Jayaraman et al., 2020). Furthermore, findings of several clinical studies support these preclinical observations by documenting that the severity of stroke and traumatic brain injury, and subsequent mortality, is associated with elevated levels of bradykinin, substance P, and neurotensin (Januzzi et al., 2016; Kunz et al., 2013; Lorente et al., 2016; Nicoli et al., 2020; Zacest et al., 2010).

## 5 NLN IN DEGRADATION OF BRADYKININ, SUBSTANCE P, AND NEUROTENSIN

Nln (EC 3.4.24.16) is a zinc endopeptidase from the same family of metallopeptidases (M3 family) as ACE, ACE2 and neprilysin (Checler & Ferro, 2018; Dauch et al., 1995), and is best known for hydrolysis of several extracellular bioactive peptides including bradykinin, neurotensin and substance P (Checler et al., 1995; Shrimpton et al., 2002; Wangler et al., 2012, 2016). Experimental studies have documented altered expression of Nln in several disease conditions including stroke (Checler, 2014; Rashid et al., 2010, 2014), and suggested its cerebroprotective function via inactivation of bradykinin, substance P, and neurotensin, and formation of angiotensin‐(1‐7) and enkephalins (Jayaraman et al., 2020; Karamyan, 2019). In a stroke setting, pharmacological inhibition of Nln after ischemia was accompanied by aggravated outcomes (brain infarction, edema formation, BBB impairment, and neuroinflammation) and elevated levels of all three peptides, whereas viral vector‐driven upregulation of Nln before stroke was associated with cerebroprotection and decreased levels of bradykinin, substance P, and neurotensin (Jayaraman et al., 2020; Karamyan, 2021).

It is noteworthy, that within the context of the RAS, the role of Nln in conversion of angiotensin I to angiotensin‐(1‐7) is not well recognized (Karamyan & Speth, 2007a; Wangler et al., 2016). This is despite experimental data pointing out to ~5‐fold higher affinity of angiotensin I for Nln versus ACE, K_i_ values 5.35 µM versus 25.88 µM (Rioli et al., 2003), and ~2‐fold higher catalytic efficiency (k_cat_/K_m_ value) of angiotensin I hydrolysis by Nln versus ACE–3.0 x 10^5^ M^−1 ^s^−1^ versus 1.8 × 10^5^ M^−1 ^s^−1^ (Rice et al., 2004; Rodd & Hersh, 1995). However, hydrolysis of angiotensin II by Nln is debatable, because some studies support inactivation of this peptide (cleavage of Tyr^4^‐Ile^5^ bond) by Nln (Dahms & Mentlein, 1992; Rioli et al., 2003), while others do not (Barelli et al., 1993; Vincent et al., 1996).

In regard to potential relevance to COVID‐19, Nln is the only other peptidase, in addition to ACE and neprilysin that has been linked to both inactivation of these three vasoactive peptides (bradykinin, substance P, and neurotensin) and microvascular permeability, edema formation, neuroinflammation, and stroke. Since altered expression of Nln has been documented in several disease conditions including stroke (Rashid et al., 2014), Alzheimer's disease (Teixeira et al., 2018), Parkinson's disease (Plum et al., 2020), and certain cancers (Mirali et al., 2020), it is plausible to suggest that expression of Nln is likely to be changed in COVID‐19 patients. Given that Garvin and colleagues (Garvin et al., 2020) have documented decreased expression of ACE and neprilysin in COVID‐19 patients, and expected elevated levels of bradykinin (i.e. inefficient degradation of the peptide), then it is likely that the expression of Nln is also decreased in these patients (otherwise, it could efficiently inactivate bradykinin).

## 6 CONCLUDING REMARKS

The original hypotheses proposed by van de Veerdonk et al. (van de Veerdonk et al., 2020) and Roche and Roche (Roche & Roche, 2020), and subsequent preliminary experimental confirmation and proposed mechanistic model by Garvin and colleagues (Garvin et al., 2020) placing bradykinin, that is, “bradykinin storm” hypothesis, in the center of COVID‐19 pathology is scientifically intriguing and important for better understanding of this complex disease. Based on their new gene expression analysis data, Garvin and colleagues (Garvin et al., 2020) elegantly link the decreased expression of ACE to potential elevation of bradykinin levels and subsequently, to many of COVID‐19 outcomes. Furthermore, the observed upregulation of bradykinin receptors and enzymes involved in its generation support the case for these hypotheses. Unfortunately, the presented picture may not be complete, since these investigators did not account for two other major peptidergic systems, substance P and neurotensin, which are also inactivated by ACE and neprilysin (both downregulated in COVID‐19 patients). Both peptides are well‐recognized to be potent mediators of microvascular permeability, edema formation and inflammation—mechanisms that are in the core of the “bradykinin storm” hypothesis. Furthermore, it is likely that another key peptidase, Nln is also affected in COVID‐19 patients, since it is the only other enzyme that inactivates all three peptides (bradykinin, substance P, and neurotensin) and has been associated with stroke, brain edema formation, increased brain microvascular permeability and neurogenic inflammation (part of COVID‐19‐associated outcomes). The case for Nln is perhaps more strengthened by lack of strong evidence that angiotensin receptor blockers (ARBs) are better tolerated in COVID‐19 patients than ACE inhibitors (these drugs would further lower activity of ACE and hence result in increased levels of bradykinin) (Sparks et al., 2020; Speth, 2020). Moreover combined use of a neprilysin inhibitor with an ARB (sacubitril/valsartan) in COVID‐19 patients is currently being investigated (Acanfora et al., 2020), although an opposing hypothesis (i.e., protective function of neprilysin) has also been suggested for COVID‐19 therapy (Mohammed El Tabaa & Mohammed El Tabaa, 2020).

Additional studies, including extended analysis of the gene expression data from Garvin et al. study (Garvin et al., 2020), could shed light on whether substance P and neurotensin (including their precursors and receptors) and peptidase Nln might also be affected and hence be part of the mechanisms explaining the pathology of COVID‐19. This is critical since bradykinin, substance P, and neurotensin may potentiate each other's effects throughout the body similar to their effect on permeability of BBB (Al‐Ahmad et al., 2021). Notably, while interpreting gene expression data (Garvin et al., 2020), it is critical to recognize that neither the expression levels of the target proteins (precursors, enzymes, receptors), nor activity of these enzymes are being evaluated. For example, it is expected that SARS‐CoV‐2, similar to its counterpart SARS‐CoV (Kuba et al., 2005), downregulates ACE2 during internalization to the host cell. However, gene expression data in COVID‐19 patient samples indicates upregulation (199‐fold) of this peptidase (Garvin et al., 2020). Evaluation of ACE2 protein expression and enzymatic activity could clarify this question, since downregulated ACE2 would lead to accumulation of des‐Arg^9^‐bradykinin, endogenous bradykinin B_1_ receptor agonist, (Guy et al., 2003) and be beneficial for the proposed “bradykinin storm” hypothesis. Importantly, ACE2 does not hydrolyze bradykinin, substance P, and neurotensin (Guy et al., 2003; Vickers et al., 2002), and hence changes in its expression levels would not directly alter availability of these peptides. Furthermore, measurement of peptide levels following proper sampling procedures (Basu et al., 2015; Karamyan et al., 2009; Karamyan & Speth, 2007b) is important for complete delineation of the proposed mechanisms. Such inclusive approach should allow to better understand the potential interactions of these peptidergic systems with the RAS in COVID‐19 patients, and enhance our understanding of viable therapeutic interventions as well as their possible adverse side effects. Whether ACE polymorphism and similar unrecognized features of neprilysin and Nln may play a role in these mechanisms is also important to consider. Lastly, it is critical to recognize that bradykinin, substance P, and neurotensin can be metabolized by other enzymes (e.g., thimet oligopeptidase, prolyl endopeptidase, dipeptidyl peptidase‐4), and that ACE, neprilysin and Nln process some other bioactive peptides.

In summary, based on the above‐discussed evidence, it is likely that not just bradykinin but also substance P and neurotensin are responsible for many of COVID‐19 outcomes. If proved by additional data analyses and new studies, it could mean that “vasoactive peptide storm,” that is, bradykinin, substance P, and neurotensin together, are the major drivers of increased microvascular permeability and inflammation‐induced complications of COVID‐19 pathology. Confirmation of this “vasoactive peptide storm” hypothesis could mean that simultaneous inhibition of all three peptidergic systems would be therapeutically more advantageous rather than modulation of any single mechanism alone. In this regard, the therapeutic potential of recombinant neurolysin (Karamyan, 2021; Wangler et al., 2016) and neprilysin (Mohammed El Tabaa & Mohammed El Tabaa, 2020; Walker et al., 2013), or small molecule activators of these peptidases may be considered (Karamyan, 2019; Kuruppu et al., 2016) while carefully evaluating their systemic effects.

## CONFLICTS OF INTEREST

VTK is the senior inventor on a patent (PCT/US2019/048702), filed by Texas Tech University Systems, focusing on discovery of small molecule activators of Nln.

## AUTHOR CONTRIBUTION

VTK conceived and wrote the paper.
